# dbCAN-seq update: CAZyme gene clusters and substrates in microbiomes

**DOI:** 10.1093/nar/gkac1068

**Published:** 2022-11-18

**Authors:** Jinfang Zheng, Boyang Hu, Xinpeng Zhang, Qiwei Ge, Yuchen Yan, Jerry Akresi, Ved Piyush, Le Huang, Yanbin Yin

**Affiliations:** Nebraska Food for Health Center, Department of Food Science and Technology, University of Nebraska, Lincoln, NE 68588, USA; School of Computing, University of Nebraska, Lincoln, NE 68588, USA; Nebraska Food for Health Center, Department of Food Science and Technology, University of Nebraska, Lincoln, NE 68588, USA; School of Computing, University of Nebraska, Lincoln, NE 68588, USA; Nebraska Food for Health Center, Department of Food Science and Technology, University of Nebraska, Lincoln, NE 68588, USA; Nebraska Food for Health Center, Department of Food Science and Technology, University of Nebraska, Lincoln, NE 68588, USA; Department of Statistics, University of Nebraska, Lincoln, NE 68588, USA; Curriculum in Bioinformatics and Computational Biology, University of North Carolina at Chapel Hill, NC, USA; Nebraska Food for Health Center, Department of Food Science and Technology, University of Nebraska, Lincoln, NE 68588, USA

## Abstract

Carbohydrate Active EnZymes (CAZymes) are significantly important for microbial communities to thrive in carbohydrate rich environments such as animal guts, agricultural soils, forest floors, and ocean sediments. Since 2017, microbiome sequencing and assembly have produced numerous metagenome assembled genomes (MAGs). We have updated our dbCAN-seq database (https://bcb.unl.edu/dbCAN_seq) to include the following new data and features: (i) ∼498 000 CAZymes and ∼169 000 CAZyme gene clusters (CGCs) from 9421 MAGs of four ecological (human gut, human oral, cow rumen, and marine) environments; (ii) Glycan substrates for 41 447 (24.54%) CGCs inferred by two novel approaches (dbCAN-PUL homology search and eCAMI subfamily majority voting) (the two approaches agreed on 4183 CGCs for substrate assignments); (iii) A redesigned CGC page to include the graphical display of CGC gene compositions, the alignment of query CGC and subject PUL (polysaccharide utilization loci) of dbCAN-PUL, and the eCAMI subfamily table to support the predicted substrates; (iv) A statistics page to organize all the data for easy CGC access according to substrates and taxonomic phyla; and (v) A batch download page. In summary, this updated dbCAN-seq database highlights glycan substrates predicted for CGCs from microbiomes. Future work will implement the substrate prediction function in our dbCAN2 web server.

## INTRODUCTION

CAZymes (Carbohydrate Active EnZymes) are enzymes that primarily target glycosidic linkages to degrade, synthesize or modify all carbohydrates on Earth (1). CAZymes are very abundant in plants and plant-associated microbes (2). For example, the human gut is a carbohydrate rich environment with a very high diversity of carbohydrate-degrading bacteria. The combined CAZyme repertoire of the gut microbiome numbers into tens of thousands of added genes (3). CAZymes are extremely important to research in human health, nutrition, gut microbiome, bioenergy, plant disease and global carbon recycling.

We published the dbCAN-seq database in 2018 (2) to provide pre-computed CAZyme and CGC (CAZyme gene cluster) sequence and annotation data for 5349 bacterial isolate genomes. CGC is a term that we have defined to describe physically linked CAZyme-containing gene clusters in microbial genomes (2). In addition to CAZymes, CGCs must also contain at least one of the other three types of signature genes: transporters (TCs), signal transduction proteins (STPs) and transcriptional factors (TFs). All these proteins/enzymes work together to assemble into a pipeline for the substrate processing and transportation, enabling an efficient complex carbohydrate degradation. In the biochemical literature, PUL (polysaccharide utilization loci) (4) is a more popular term, describing gene clusters utilizing complex carbohydrate substrates, e.g. XUL (xylan utilization loci), ChiUL (chitin utilization loci), and XyGUL (xyloglucan utilization loci) (5–7). PULs were originally described in Bacteroidetes and also present in other gram-negative bacteria (e.g. Proteobacteria), and contain the hallmark SusC/D transport system (8). More recently, PULs were also identified in gram-negative bacteria such as Firmicutes and Actinobacteria, which use ABC transporters and substrate-binding proteins (SBP) instead of the SusC/D transport system (4). Hundreds of PULs have been biochemically characterized targeting almost all major carbohydrate substrates and assembled into the PULDB (9) and dbCAN-PUL (10) databases. Essentially, PULs are experimentally verified CGCs with characterized glycan substrates.

Although originally developed for CAZymes and CGCs in bacterial isolate genomes, dbCAN-seq has been frequently cited by papers studying environmental microbial communities, e.g. animal gut and soil microbiomes (11–15). In the past 5 years, numerous microbiomes have been sequenced and hundreds of thousands of metagenome assembled genomes (MAGs) from various ecological environments are now available in the public databases such as the MGnify database of European Bioinformatics Institute (16) and the IMG/M database of Joint Genome Institute (17). Currently, no databases collect CAZymes and CGCs from microbiome MAGs and provide them on the web.

In the meantime, the CAZyme bioinformatics field continues to develop. It is now possible to infer carbohydrate substrates for CAZymes and CGCs, which is of a huge interest to applied microbiome (18,19). For instance, the microbiome-based personalized nutrition aims to use individualized dietary intervention strategy to modulate human gut microbiomes for improved human health (20). The ability to predict what prebiotic glycans that a patient's gut microbiome might respond to will be highly useful to dieticians and nutritionists (21) to make individualized dietary recommendations. Importantly, we have also frequently received requests from users of our dbCAN web server (22,23) to develop a function to predict glycan substrates for given MAGs of various microbiomes. Clearly, the glycan substrate is a highly useful information to be included in dbCAN-seq.

In this dbCAN-seq database update, we have made two major and significant advances: (i) dbCAN-seq now provides a comprehensive CAZyme and CGC catalog from microbiomes of four ecological environments (human gut, human oral, cow rumen, marine); (ii) dbCAN-seq uses two novel approaches to the prediction of substrates for microbiome CGCs, and provides a browse by substrate function to allow the search of CGCs targeting specific substrates that are predicted in different microbiomes. It should be noted that all predicted substrate assignments for CGCs in dbCAN-seq need experimental validation. It is our hope that these predicted CGCs and substrates in the microbiomes of four ecological environments could facilitate the experimental characterization of new PULs by the carbohydrate community.

## DATABASE CONTENT

### Collecting CAZymes and CGCs from four datasets of MAGs

We included four MAG datasets from the EBI MGnify (16) database. Each dataset contains genomes of thousands of prokaryotic species, and each species often have multiple MAGs. For example, the human gut dataset (also known as the Unified Human Gastrointestinal Genome [UHGG] collection) contains 289 231 genomes (96.2% are MAGs and the rest are isolate genomes) of 4744 species (Table 1). However, we only included the representative genome (usually the one with the best quality) of each species (i.e. one genome per species) to reduce data redundancy in dbCAN-seq.

**Table 1.** CAZyme and CGC data statistics in dbCAN-seq

MAG databases	Human gut	Human oral	Cow rumen	Marine	Total
Total # of MAGs *	4744	452	2729	1496	9421
High-quality MAGs	3329	246	1479	726	5780
Total # of proteins	10 231 988	787 607	5 343 176	3 489 050	19 851 821
# of CAZymes	261 386	17 899	164 889	53 872	498 046
% of CAZymes	2.55%	2.27%	**3.09%**	1.54%	2.51%
# of MAGs	4741	449	2720	1491	9401
% of MAGs	99.94%	99.34%	99.67%	99.67%	99.79%
# of CGCs	94 276	6 131	50 132	18 367	168 906
# of MAGs	4688	440	2704	1475	9307
% of MAGs	98.82%	97.35%	**99.08%**	98.60%	98.79%
# of CAZymes	146 039	9 010	74 934	24 510	254 493
% of CAZymes	55.87%	50.34%	**45.45%**	45.50%	51.10%

*We only used the representative genomes of each species. High-quality MAGs have genome completeness rate > 90% and contamination rate < 5%.

**Figure 1.**
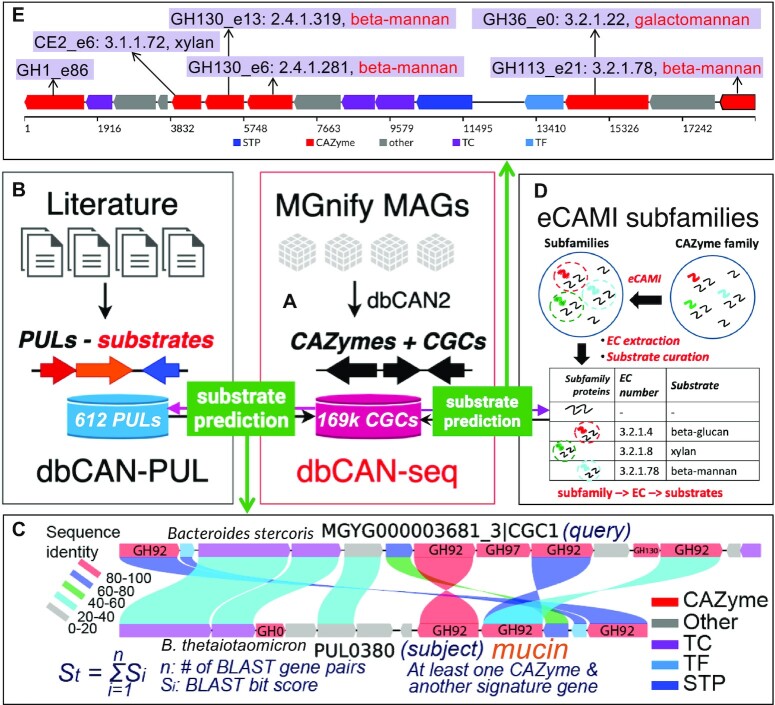
Overview of the update of dbCAN-seq. (**A**) Four MAG datasets of MGnify are processed by the run_dbcan program of dbCAN2 for CAZymes and CGCs. CGCs are then subject to substrate predictions using two approaches. (**B**) dbCAN-PUL contains 612 PULs and their substrates curated from literature. A PUL → substrate mapping table is built (Supplementary Table S1). (**C**) An example alignment of CGC (query) and PUL (subject) shows all the protein pairs sharing significant sequence identity. The four signature gene classes (CAZyme, TC, TF, STP) and the ‘Other’ class are colored differently. The best PUL hit for the query is determined by the summed BLAST bit scores of all protein pairs, which must contain at least one CAZyme and one other signature genes. The best PUL hit's substrate (from Supplementary Table S1) is assigned to the query CGC. (**D**) eCAMI precomputed CAZyme subfamilies are examined to extract EC numbers from experimentally characterized proteins (from CAZyDB). From the eCAMI subfamily and EC numbers, substrates are manually curated from the CAZyDB and assigned to the subfamily. A CAZyme subfamily → EC → substrate mapping table is built (Supplementary Table S2). (**E**) An example CGC has been annotated by dbCAN2. The CAZymes are labeled with their eCAMI subfamilies, EC numbers, and curated substrates (from Supplementary Table S2). Not all eCAMI subfamilies have EC numbers (no experimentally characterized proteins in the subfamily) and thus have no assigned substrates. The CGC’s substrate is inferred to be the substrate assigned to the majority of CAZymes.

The *run_dbcan* program of dbCAN2 (23) was run on all the MAGs to predict CAZymes and CGCs with default parameters (Figure 1A). Specifically, CAZymes were predicted by three tools (HMMER versus dbCAN-HMMdb (22), DIAMOND versus CAZyDB (1), eCAMI versus precomputed CAZyme peptide library (24), all packaged into *run_dbcan*) and those predicted by ≥2 tools were kept. CGCs were predicted with the CGC-Finder program (packaged into *run_dbcan*) with CAZyme + TC + TF option and ≤2 inserted non-signature genes option (see dbCAN2 website for details). The non-signature genes were further extracted for Pfam annotation.

Table 1 shows that close to half of a million of proteins were annotated as CAZymes. On average 2.51% of the total number of genes in the four MAG datasets encode CAZymes. Over 51% of the CAZymes are clustered with other signature genes (TC, TF, STP) forming ∼169 000 CGCs. Comparing the four MAG datasets, cow rumen MAGs have the highest percentage of CAZymes (3.09%), twice as much as marine MAGs (1.54%). Cow rumen also has the highest percentage of MAGs encoding CGCs (99.08%) compared to 97.35% in human oral MAGs. Human gut MAGs have the highest percentage of CAZymes (55.87%) present in CGCs, while unexpectedly cow rumen has the lowest percentage of CAZymes (45.45%) present in CGCs. This could be due to two factors: (i) cow rumen MAGs have lower genome assembly quality (e.g. more fragmented and incomplete) than human gut MAGs; (ii) cow rumen is inhabited by more bacteria that employ other mechanisms for carbohydrate degradation (e.g. cellulosomes, free enzymes, type 9 secretion systems, and outer membrane vesicles) (5–7) than the classical gene clustering mechanism to form PULs.

### Predicting carbohydrate substrates for CGCs

Once the CGCs were predicted, we developed two computational approaches to infer their glycan substrates. Two key glycan substrate mapping files were prepared by manual curation of: (i) the dbCAN-PUL database (10) to obtain the PUL → substrate mapping (Supplementary Table S1) and (ii) the CAZyme subfamilies of eCAMI (24) and the characterized proteins of CAZyDB (1) to obtain the CAZyme subfamily → EC → substrate mapping (Supplementary Table S2).

The first approach is based on the BLAST comparison (25) of protein sequences of the query CGCs (Figure 1A) against the protein sequences of the 612 PULs of dbCAN-PUL (Figure 1B). All the 612 PULs have known substrates curated from literature (Supplementary Table S1). An example is shown in Figure 1C, where the query CGC has nine proteins sharing significant sequence similarity to proteins of the subject PUL (with known substrate mucin). One query CGC could have multiple PUL hits in dbCAN-PUL. The best hit was selected with the highest summed BLAST scores (Figure 1C) of all matches between the query and the subject. It is also required that there are at least one CAZyme match plus at least one match from one of the other signature gene categories (i.e. TC, STP, TF). The best PUL hit's substrate will be assigned to the query CGC.

The second approach is based on the inspection of the eCAMI subfamily (Figure 1D) annotated substrates of all component CAZymes in the CGC. Figure 1E shows an example CGC, which contains 6 CAZymes annotated by *run_dbcan*. One of the three component tools integrated in *run_dbcan* is eCAMI (24), which can not only assign a protein to a CAZyme family but also to a subfamily (e.g. GH130_e13, Figure 1E). The eCAMI subfamily is precomputed for all CAZyme families and the subfamily name contains an ‘e’ to distinguish it from the existing CAZyDB subfamilies (e.g. GH5_1), which are currently available for only 27 CAZyme families. The correspondence between eCAMI subfamilies and CAZyDB subfamilies of 24 CAZyme families has been studied in our previous paper (24), which showed the great accuracy and purity of eCAMI subfamilies matching CAZyDB subfamilies (Figure 1 of (24)).

Briefly, eCAMI is an amino acid k-mer based CAZyme annotation tool. It has two functions: (i) classify all proteins of each CAZyme family (assigned by CAZyDB) into subfamilies and extract the distinguishing k-mer peptides of each subfamily; (ii) predict the CAZyme family and subfamily membership for a query protein according to the presence of the distinguishing k-mer peptides. Due to the poly-specificity, one CAZyme family can contain proteins of multiple EC numbers: e.g. GH5 contains experimentally characterized proteins of over 20 different EC numbers targeting over 10 different carbohydrate substrates. Classifying proteins into subfamilies (Figure 1D) can partially address the poly-specificity issue, as many subfamilies can have much fewer or even one single specific EC number. Once the eCAMI subfamily and EC number is determined for a CAZyme protein, the CAZyme subfamily → EC → substrate mapping table (Supplementary Table S2) is looked up to assign a substrate for the protein (Figure 1E). Using a simple majority voting rule, a substrate assignment for a CGC can be inferred by considering all the CAZymes in the CGC and their substrates.

### Overview of CGCs with predicted carbohydrate substrates

**Figure 2.**
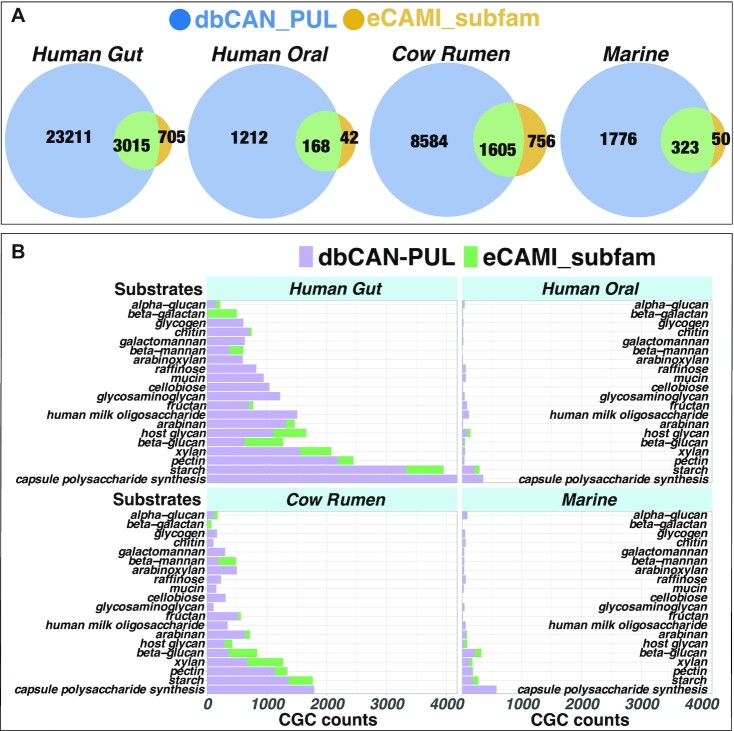
Overview of CGC counts with predicted substrates in dbCAN-seq. (**A**) The venn diagrams of CGCs annotated by the dbCAN-PUL approach and by the eCAMI subfamily approach. The intersections are CGCs annotated by both approaches (available in Supplementary Table S3). (**B**) The top 20 most abundant substrate groups. The x-axis is the CGC count. The y-axis shows the substrate groups.

Table 2 shows that the first approach (dbCAN-PUL) predicted substrates for 40 574 (24.02%) CGCs in the four MAG datasets. The second approach (eCAMI subfamily) only predicted substrates for 6664 (3.95%) CGCs. These low percentages are not surprising given that the theoretical carbohydrate structural diversity in nature is astonishingly high (26). The un-annotated CGCs likely target those unknown carbohydrates in the four environments. Together the two approaches predicted substrates for 41 447 (24.54%) CGCs (the sum of all areas in Figure 2A), among which 5111 CGCs have substrate assignments (12.33%) by both approaches (the sum of all intersections in Figure 2A). The substrate assignments of the two approaches agree in 4183 (81.8%) of the 5111 CGCs (Supplementary Table S3), which are more reliable predictions. This percentage is likely underestimated due to the complex structures of carbohydrates (27) and the inconsistent, non-standard, and mixed use of glycan nomenclature in the literature (28). For example, pectins have alpha-rhamnoside and beta-galactan side chains, which are also found in arabinogalactans (27); beta-galactan also exists in algal polysaccharides such as carrageenans (7,27).

**Table 2.** CGC-substrate data statistics in dbCAN-seq

MAG databases	Human gut	Human oral	Cow rumen	Marine	Total
# of CGCs with predicted substrates (via dbCAN-PUL)	26 846	1407	10 222	2099	40 574
% of CGCs	**28.48%**	22.95%	20.39%	11.43%	24.02%
# of MAGs	3841	339	2089	742	7011
% of MAGs	**80.97%**	75.00%	76.55%	49.60%	74.42%
# of CAZymes	65 589	3 218	25 999	5 605	100 411
% of CAZymes (out of total # of CAZymes in CGCs)	**44.91%**	35.72%	34.70%	22.87%	39.46%
# of CGCs with predicted substrates (via eCAMI subfamily)	3720	210	2361	373	6664
% of CGCs	3.95%	3.43%	**4.71%**	2.03%	3.95%
# of MAGs	1 611	115	945	192	2 863
% of MAGs	33.96%	25.44%	**34.63%**	12.83%	30.39%
# of CAZymes	15 089	698	9436	1557	26 780
% of CAZymes (out of total # of CAZymes in CGCs)	10.33%	7.75%	**12.59%**	6.35%	10.52%

In all four MAG datasets, the dbCAN-PUL approach predicted substrates for more CGCs (larger blue areas than yellow areas in Figure 2A) from more MAGs (% of MAGs row in Table 2) than the eCAMI subfamily approach. For example, in human gut MAGs, 26,846 CGCs (from 80.97% MAGs) were assigned with a substrate by the dbCAN-PUL approach, compared to 3,720 CGCs (from 33.96% MAGs) by the eCAMI subfamily approach. It is interesting that a majority of eCAMI subfamily predicted CGCs are also covered by the dbCAN-PUL approach.

Comparing the four MAG datasets, human gut and cow rumen have higher percentages of CGCs and MAGs with a predicted substrate than human oral and marine datasets (Table 2). This is expected because animal guts have a high density of complex carbohydrates feeding highly efficient carbohydrate degraders, which are equipped with a great variety of molecular machineries for carbohydrate degradation (5–7). Another possible reason is that the current CAZy database and dbCAN-PUL databases are biased towards CAZymes and PULs from gut bacteria. For example, the most published PULs in the past five years are characterized from gut bacteria of humans and other animals (10).

Figure 2B shows a breakdown of CGCs according to the top 20 predicted substrates. Capsular polysaccharide (CPS) synthesis CGCs were predicted solely by the search against dbCAN-PUL (purple bars), which contains 117 PULs for O-antigen and CPS synthesis. These PULs/CGCs often contain GT (glycosyltransferase) families of CAZymes (e.g. GT2 and GT4). Strictly speaking they are biosynthetic gene clusters (BGCs) instead of CGCs/PULs. Starch, pectin, and xylan are the top three most abundant CGC groups in both human gut and cow rumen. In human oral MAGs, the top three groups are starch, host glycan, and human milk polysaccharide, while in marine MAGs, beta-glucan (e.g. laminarin of brown algae) is the highest and starch is the second.

## WEB DESIGN

**Figure 3.**
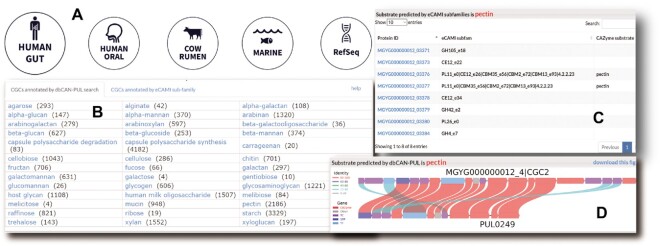
Screenshots of dbCAN-seq website. (**A**) Choose from five databases in Home page. (**B**) Browse by substrate in Home page. (**C**) eCAMI subfamily table to support substrate prediction. (**D**) CGC vs PUL alignment to support substrate predicted by dbCAN-PUL.

The dbCAN-seq website (https://bcb.unl.edu/dbCAN_seq) uses the same web framework and template as the previous version. From the Home page, users can select which dataset (RefSeq, human gut, human oral, cow rumen, and marine) for data browse and search. Selecting RefSeq will go to the old dbCAN-seq website (https://bcb.unl.edu/dbCAN_seq_old), while selecting the four MAG datasets will load all the data of the chosen environment for web browsing (e.g. human gut is selected in Figure 3A).

### Browse by substrate

In addition to the CAZyme and CGC data from four MAG datasets, the dbCAN-seq website has a browse by substrate function via: (i) the Home page (click on a substrate, Figure 3B), (ii) the Statistics page (click on the substrate summary bar plot) and (iii) the Search engine (use a specific substrate, e.g. starch, as keyword to search).

### CGC page

Compared to the old website, we now provide more graphs in the CGC page: (i) the CGC gene composition diagram (a graph like Figure 1E) and (ii) the diagram of CGC versus PUL alignment for substrate predicted by dbCAN-PUL (Figure 3D) if exists. An example is at https://bcb.unl.edu/dbCAN_seq/cgc_one_new.php?cgc_id = MGYG000000012_4|CGC2. The CGC page also contains a gene composition table, an eCAMI subfamily table to list all CAZymes to support substrate predicted by eCAMI subfamilies (Figure 3C), and the Jbrowse browser.

### Download page

Users can now batch download all the CAZymes and CGCs in tsv summary and fasta sequence formats as compressed tarballs. The substrate predictions for CGCs are also organized for each substrate group and available to download as tsv files.

### Statistics page

This page is designed to provide detailed statistics of all the CAZyme, CGC and substrate data. It is organized into five tables: (i) gene statistics table, (ii) CGC statistics table, (iii) genes in CGCs statistics table, (iv) table of gene statistics of annotated CGCs (with substrate), (v) MAG statistics table. Data are also organized as bar graphs including: (i) distribution of CGCs by substrate predicted by dbCAN-PUL search, (ii) distribution of CGCs by substrate predicted by eCAMI sub-family, (iii) counts of CGCs with the plotted PUL as the best hit from dbCAN-PUL, (iv) counts of CGC-encoding MAGs in each phylum. Clicking on each bar will open a table for a more organized and convenient data browsing.

## FUTURE WORK

We plan to update dbCAN-seq annually to include more MAG datasets from more ecological environments, e.g. gut MAGs from mouse, pig, goat, as well as MAGs from soils and the earth microbiome project. The two substrate prediction approaches implemented in the current dbCAN-seq database will be supplemented by more advanced supervised machine learning methods to predict substrates for more CGCs. Unsupervised machine learning approaches will also be explored to infer novel CGC families that target uncharted glycan substrates in nature. In summary, dbCAN-seq is an important component of our popular dbCAN tool suite, which also contains dbCAN2 (22,23), CGC-Finder (23), eCAMI (24) and dbCAN-PUL (10).

## DATA AVAILABILITY

All the data are free available online at https://bcb.unl.edu/dbCAN_seq.

## Supplementary Material

gkac1068_Supplemental_Files
