# Supplementary material for: dbCAN-seq update: CAZyme gene clusters and substrates in microbiomes
Source: Nucleic Acids Res. 2022 Nov 18;51(D1):D557–63. doi: 10.1093/nar/gkac1068 (PMC9825555; doi:10.1093/nar/gkac1068)
Supplement: gkac1068_Supplemental_Files [file gkac1068_supplemental_files.zip › S-TABLES.docx]

**SUPPLEMENTARY TABLES**

**Table S1**: PUL -> substrate mapping table manually curated from dbCAN-PUL database and literature

**Table S2**: CAZyme subfamily -> EC -> substrate mapping table manually curated from eCAMI subfamilies and the characterized proteins of CAZyDB

**Table S3**: 5,111 CGCs and their substrate assignments by the two approaches (4,183 are agreed)
